# Celebrating 15 years of *Chemical Science*!

**DOI:** 10.1039/d4sc90249a

**Published:** 2024-12-11

## Abstract

In 2025, *Chemical Science* celebrates its 15th anniversary, and 10 years of Diamond Open Access. Dr May Copsey, Executive Editor of *Chemical Science*, introduces the 15th anniversary of the journal.

## Abstract 2

In 2025, *Chemical Science* celebrates its 15th anniversary, and 10 years of Diamond Open Access.

## Graphical abstract

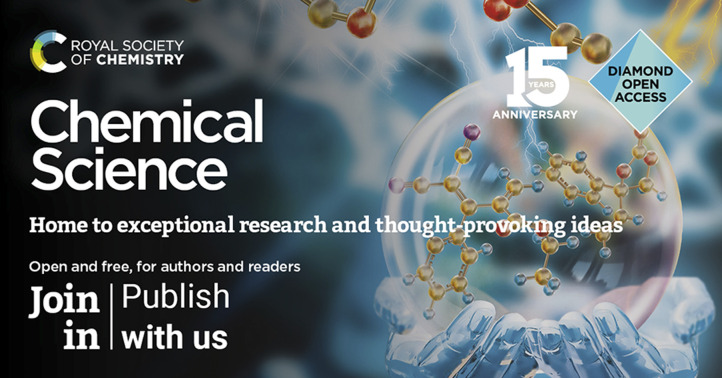

A very warm welcome to the first issue of *Chemical Science* for 2025!

This year promises to be an exciting one for the journal and we have not one, but two fantastic reasons to celebrate in 2025.

In June, the journal will celebrate 15 years of publication. The Royal Society of Chemistry (RSC) first launched *Chemical Science* back in 2010, publishing its first issue in June of that year. Since then we have seen the journal develop as the flagship journal of the Royal Society of Chemistry, aiming to publish research of exceptional significance across the breadth of the chemical sciences, and in doing so, support thousands of authors with the dissemination of their research.

## 15 years of our *Chemical Science* community

The journal of course would not be possible without the support of our many thousands of authors, reviewers, Editors and Advisory Board members during this time. In celebration of this, and our journal community in general, we will publishing a 15th Anniversary Community Collection. We hope this collection will reflect the research of our broad community, and showcase some of the most exciting research across the chemical sciences. Look out for further information as we start to compile this special collection, and we'll be highlighting articles from this in particular issues throughout 2025.

We are also working on a Leading Investigators collection for 2025. We are excited to publish and showcase research from leading investigators from across all areas of chemistry who started their independent career during the last fifteen years, or the lifetime of the journal. This is the first collection of this type in *Chemical Science*, and we have been inspired by the success of our fellow RSC journals and the numerous Emerging Investigator collections that have become such an integral part of the RSC journals portfolio.

Other exciting things to come in 2025 include a *Chemical Science* Lectureship, and the next event in the *Chemical Science* Symposia series, which are held at the home of the Royal Society of Chemistry at Burlington House. Watch out for further updates and news on these to come.

## 10 years of Diamond Open Access

2025 is also a special year as it marks 10 years of *Chemical Science* being Diamond Open Access. During this time, we have published over 13 000 articles as open access, with authors from 88 countries, and we have chosen to do this with no charges to our authors.

At the RSC, we believe that scientific knowledge should be freely accessible to everyone, regardless of their background or affiliation. As a not-for-profit publisher, we are dedicated to supporting the transition to open access in a way that best serves global and academic communities. We are really proud that making *Chemical Science* a diamond open access journal was an early way of achieving this goal, and continues to be a part of how we deliver on this today.

For the most recent International Open Access Week in 2024, the RSC was looking at the theme of Community over Commercialisation, and Sara Bosshart, our Head of Open Access, explained our thinking behind our commitment to open access.

“We believe that open access is the right thing but there's also the aspect that the publishing industry as a whole is moving towards open access. Funders are demanding open access, so the train's already left the station and it's full steam ahead – we need to adapt and change. It's no longer a question of ‘if’, but when and how do we adapt such that we can remain sustainable.

“When we made the commitment, what we were really doing, in effect, was saying ‘we want to take charge of this movement and lead the way so we can shape what the future looks like for open access.’ We don't just want to be reactive.

“Our chemistry community is at the heart of what we do, so we want to make sure we're leading the way and creating a future that best supports the research ecosystem and enables chemists to fulfil their potential – and our RSC mission as a society – of making the world a better place.”

We have also been reflecting on the Diamond open access for *Chemical Science*, and we asked some of our authors what it meant to them:

“Actually, being open access and free ensures that a diverse group of authors can publish high impact research in this journal, ensuring that every author has a chance to publish their research.”

“The free to publish and free to read policy of *Chemical Science* makes it a unique journal who doesn't discriminate based on economic restriction and it gives an impression that this is the only journal who aims in advancement of science without any limitation.”

“Obviously, with open access more people can read *Chemical Science*. It is reachable and known for its high standards. It is a great combination!”

“Overall, the open access and free to publish status of *Chemical Science* contributes to its reputation as a forward-thinking, inclusive, and influential journal within the field of chemistry.”

## Welcome to our newest Associate Editors

At *Chemical Science*, we hear from authors regularly that they want the peer-review of their articles to be handled by leading experts in their field. We have therefore this year been adding new faces to our Editorial Board, to further develop the editorial team that will be handling your manuscript through the peer-review process. Find out more below from the newest Associate Editors we are delighted to welcome to the team.
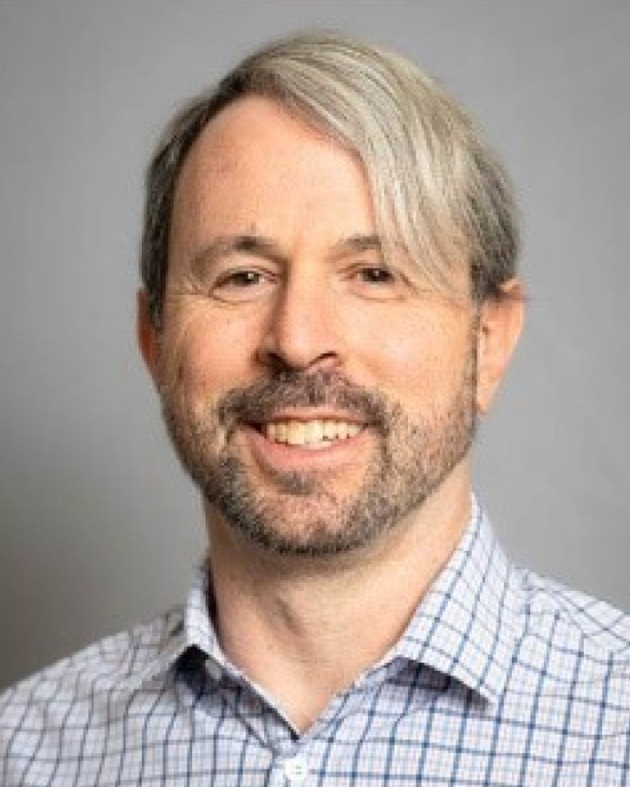


**Matt Sigman**, University of Utah

Associate Editor, organic chemistry and data-driven discovery
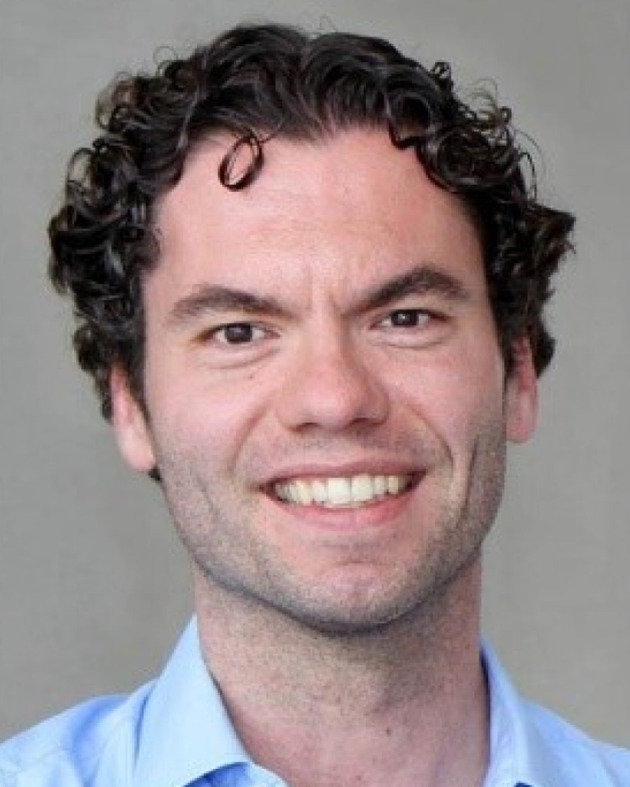


**Christian Hackenberger**, Leibniz-Institut für Molekulare Pharmakologie and Humboldt Universität zu Berlin

Associate Editor, chemical biology and organic chemistry
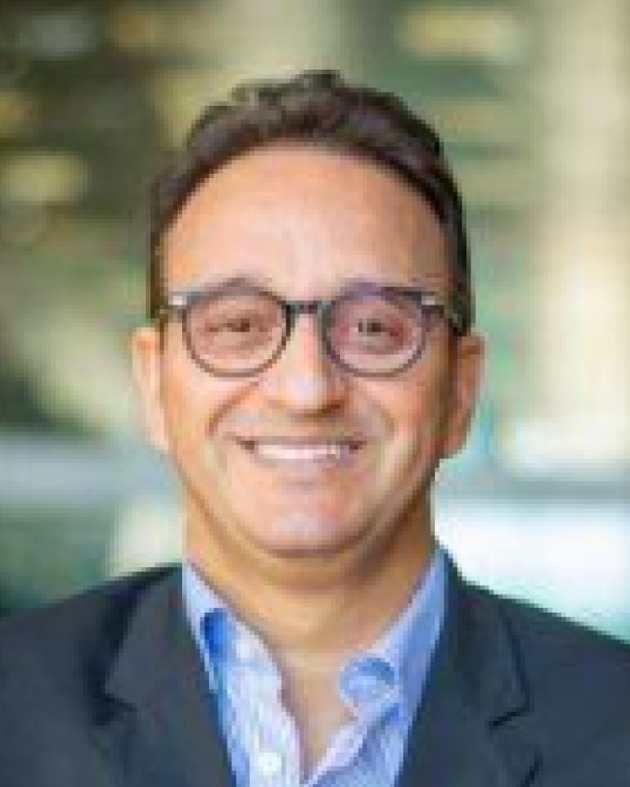


**Mohamed Eddaoudi**, King Abdullah University of Science and Technology (KAUST)

Associate Editor, Metal Organic Frameworks and other porous materials
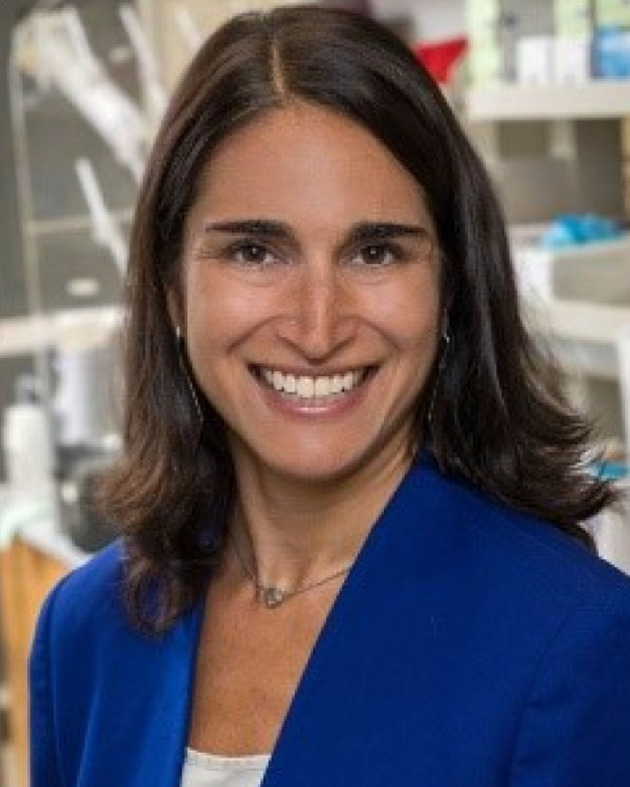


**Hannah Shafaat**, University of California, Los Angeles

Associate Editor, bioinorganic chemistry and spectroscopy
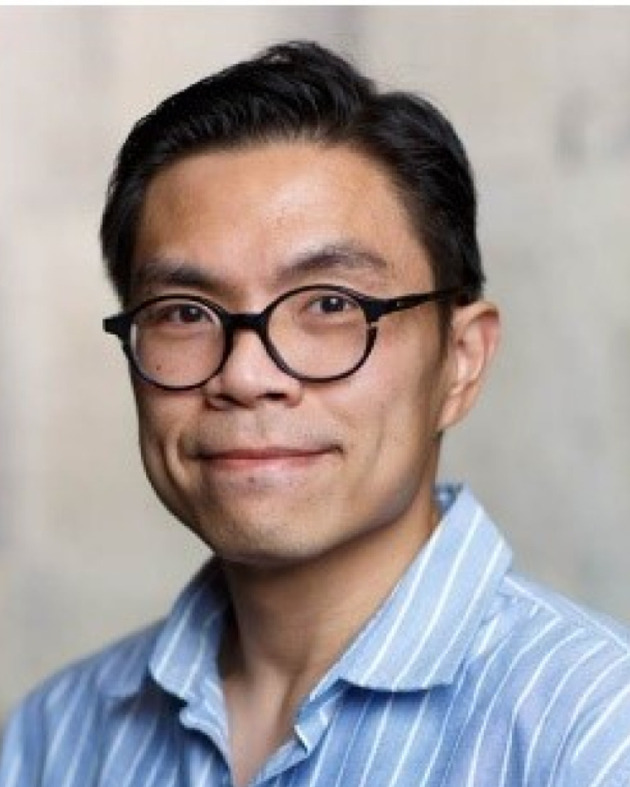


**Hans Renata**, Rice University

Associate Editor, chemical biology
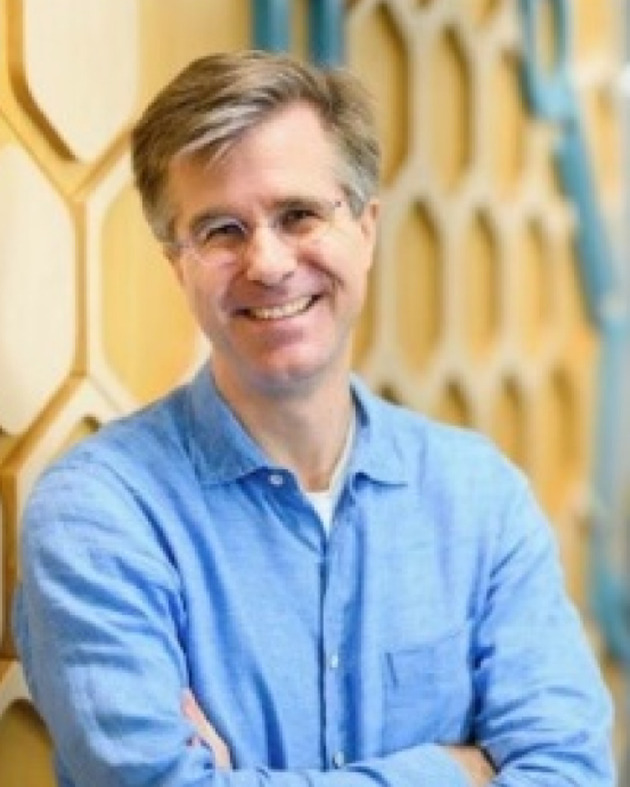


**Christopher Barner-Kowollik**, Queensland University of Technology

Associate Editor, polymer chemistry

“I always had the best experience with publishing with the RSC, especially with *Chemical Science*, regardless of the outcome. As an author I felt valued, as a referee heard and as an editor supported. If you started a fan club, I would join!”

– Christian Hackenberger

“*Chemical Science* is one of the thought leading journals in contemporary chemistry under the auspices of one of the most esteemed learned societies. Being a relatively young journal, we are dynamic and willing to publish ideas and outcomes of high quality that are not mainstream – innovation always occurs at the flanks and the out-there ideas of today will be the trend setters of the future. That's where I see *Chemical Science* playing a key role.”

– Christopher Barner-Kowollik

Finally, on behalf of the RSC team, I'd like to thank all our Editorial Board members, past and present, Advisory Board, authors and reviewers, all of whom contribute to making the journal a success. We are so grateful for all your work and support, and look forward to future success to come!

May Copsey, Executive Editor, *Chemical Science*

